# Paradoxical effect of obesity on hemorrhagic transformation after acute ischemic stroke

**DOI:** 10.1186/1471-2377-13-123

**Published:** 2013-09-23

**Authors:** Chi Kyung Kim, Wi-Sun Ryu, Beom Joon Kim, Seung-Hoon Lee

**Affiliations:** 1 Department of Neurology, Seoul National University Hospital, 101 Daehak-ro, Jongno-gu, Seoul 110-744, Republic of Korea; 2 Clinical Research Center for Stroke, Biomedical Research Institute, Seoul National University Hospital, Seoul, Republic of Korea; 3 Department of Neurology, Dongguk University Ilsan Hospital, Goyang, Republic of Korea; 4 Department of Neurology, Seoul National University Bundang Hospital, Seongnam, Republic of Korea

**Keywords:** Obesity, Hemorrhagic transformation, Bleeding-obesity paradox, Ischemic stroke, Body mass index

## Abstract

**Background:**

Among the patients with established coronary artery diseases, obese patients tend to have a more favorable prognosis, which is called as obesity paradox. Interestingly, mildly obese patients who underwent coronary revascularization had a lower risk of bleeding. In this context, we have investigated the association between obesity and hemorrhagic transformation (HTf) after acute ischemic stroke.

**Methods:**

A total of 365 patients with first-ever acute ischemic stroke were included in this study. Demographic, clinical and radiological information was collected and HTf was evaluated through follow-up T2*-weighted gradient-recalled echo MRI performed usually within 1 week after occurrence of stroke. Body mass index was calculated, and obesity was defined using the World Health Organization Western Pacific Regional Office criteria.

**Results:**

The HTf was identified in 59 patients (16.2%). As the severity of obesity increased, the occurrence of HTf decreased. Compared with the normal weight group and after controlling possible confounders including acute and previous treatment, stroke severity and subtype, the risk of HTf decreased significantly in the obese group (odds ratio, 0.39; 95% confidence interval, 0.17-0.87).

**Conclusions:**

The better outcome for HTf seen in obese patients suggests the existence of a “bleeding-obesity paradox” in acute ischemic stroke.

## Background

Hemorrhagic transformation (HTf) frequently occurs after ischemic stroke with or without thrombolytic treatment [1,2]. It was known that HTf after acute ischemic stroke was associated with poor outcome and delayed the initiation of proper anticoagulation treatment for stroke with cardioembolism [3]. Although certain studies have demonstrated the association between old age [4], high systolic pressure [5], or thrombolytic treatment [6] with HTf, the predictive factor for HTf after acute ischemic stroke is still elusive.

Obesity affects more than a billion adults worldwide, and is implicated as one of the major risk factors of cardiovascular diseases [7,8]. However, in patients with established coronary artery diseases, obese patients tend to have a more favorable prognosis, which is called as obesity paradox [8,9]. Interestingly, according to a recent study, mild obese patients who underwent coronary revascularization had a lower incidence of periprocedural bleeding, and the authors in that study called this phenomenon as “bleeding-obesity paradox” [10].

In ischemic stroke, HTf occurs after extravasation of blood over damaged cerebral vascular endothelium and is more directly associated with disease itself than bleeding after coronary revascularization [11]. However, few studies have investigated the effects of obesity on the bleeding after ischemic stroke. In this context, we sought to investigate the association between obesity and HTf, and assessed whether “bleeding-obesity paradox” exists in acute ischemic stroke.

## Methods

A total of 744 first-ever acute ischemic stroke patients who had been admitted to Seoul National University Hospital within 7 days from disease onset between October 2002 and March 2006 were consecutively enrolled in this study. According to the classification of Trial of Org 10172 in Acute Stroke Treatment, amongst the chosen patients, subjects with the following conditions were excluded from this study: transient ischemic attack (n = 79; HTf = 0), strokes attributable to small vessel disease (n = 221; HTf = 0), or strokes of other determined etiology (n = 9; HTf = 1) [12]. Patients without complete workups (n = 70; 10 without initial MRI, 37 without follow-up MRI, 14 without checking height, and 9 without laboratory workups) were further excluded. The excluded 70 patients had more severe stroke than included patients, and in-hospital death rate of excluded patients was higher than that of included patients. However, other demographic and cardiovascular risk factors including the average and the distribution of body mass index (BMI) were not different between two groups (Additional file 1: Table S1). Therefore, our study population consisted of a total of 365 patients with first-ever acute ischemic stroke. The patient or the patient’s next of kin was provided informed consent, and the study was approved by the institutional review board at Seoul National University Hospital (H-0911-065-301).

Baseline demographic and clinical information collected at admission included age at onset, gender, body weight and height at the time of admission, hypertension (a high systolic blood pressure was defined as at least 140 mmHg at discharge, and a diastolic blood pressure as at least 90 mmHg at discharge. Hypertension was defined as a high systolic blood pressure or a high diastolic blood pressure or as the current use of antihypertensive medication), diabetes (previous use of antidiabetic medication, fasting blood glucose > 7.0 mmol/L), hyperlipidemia (previous use of lipid-lowering medication, total cholesterol > 6.0 mmol/L at admission), smoking, previous use of antiplatelet or anticoagulant medications, systolic and diastolic blood pressure levels at admission, the level of blood glucose and total cholesterol at admission, the initial National Institute of Health Stroke Scale (NIHSS) score, and thrombolytic or/and acute heparin treatment during acute stage. BMI was calculated as weight (in kilograms) divided by height (in meters squared), an obesity was defined using the criteria of the World Health Organization (WHO) Western Pacific Regional Office, which reflects a different risk factors and body fat distribution in the Asian Population [13].

All the participants underwent initial brain MRI before the initiation of thrombolytic or antithrombotic therapy (within 24 hours after admission) and follow-up brain T2*-weighted gradient-recalled echo (GRE) MRI usually within 1 week after occurrence of stroke (follow-up days: mean ± SD, 6.7 ± 1.4). The MRI studies were performed using 1.5-Tesla superconducting magnet (GE Healthcare, Chalfont St. Giles, UK). The standardized MRI protocol consisted of axial T2-weighted spin echo (repetition time/echo time, 2500 to 4500/80 to 112 ms; flip angle, 20˚; slice thickness, 5 mm; gap width, 2 mm) and diffusion-weighted imaging (repetition time/echo time, 4000/73 ms; flip angle, 90˚; slice thickness, 5 mm; gap width, 2 mm). The GRE images were obtained in the axial plane with the following parameters: repetition time/echo time, 500/15 ms; flip angle, 26°; matrix size, 256 × 192; slice thickness, 6 mm; and gap width, 2 mm. The HTf was identified when follow-up GRE images showed a low-signal area consistent with blood within the acute ischemic lesion, according to the pre-specified criteria [14]. White matter lesions (WMLs) were judged as being absent, or present as punctuate, early confluent or confluent abnormalities as seen on T2-weighted MR images, according to the previously proposed method [15]. Early confluent or confluent lesions were designated as advanced WMLs in this study. Microbleeds were defined as focal homogenous areas with a diameter of 2 to 5 mm as previously described [16,17].

The χ^2^ test and the Student *t* test were used to compare categorical data and continuous data of the subjects, respectively. The distribution of demographic, clinical, and radiological variables were analyzed using χ^2^ test for trends in proportion. The odds ratio (OR) and 95% confidence interval (CI) for the HTf in each obesity status were calculated using binary logic regression analyses. For multivariable analyses, potential confounders were adjusted such as age, gender, hypertension, diabetes, hyperlipidemia, current smoking, initial NIHSS, thrombolysis, acute heparin treatment, previous use of antiplatelet or anticoagulant, stroke subtype, and the presence of advanced WMLs and microbleeds. The adjusted OR and 95% CI for the HTf or cerebral microbleeds were calculated by multivariable analysis. Probability values were 2-tailed, and P values < 0.05 were considered significant. All statistical analyses were performed using SPSS 19.0 (SPSS Inc., Chicago, IL).

## Results

Among 365 subjects, there were 246 men and 119 women. Subjects’ age ranged from 16 to 95 years, and their mean age was 64.7 years. Average BMI of subjects was 24.1 kg/m^2^. According to the WHO obesity criteria for the Asian-Pacific Population, 94 patients (25.8%) were classified as overweight (BMI, 23.0-24.9 kg/m^2^) and 146 (40%) were classified as obese (BMI, ≥ 25.0 kg/m^2^).

The HTf was noted in 59 patients (16.2%). Table 1 shows that BMI was significantly lower in subjects with HTf (23.1 kg/m^2^) that in those without HTf (24.2 kg/m^2^). The average of initial NIHSS score and the proportions with cardioembolism and thrombolytic treatment were greater in subjects with HTf. However, the average age, glucose level, cholesterol level, and systolic and diastolic blood pressure at admission, and the proportions of gender, hypertension, diabetes, hyperlipidemia, smoking, previous use of antiplatelet agents and anticoagulation, acute heparin treatment, and the presence of advanced WMLs and microbleeds were not different between those with and without HTf. As the severity of obesity increased, the incidence of HTf decreased, and the average of initial NIHSS score also decreased (Table 2). The proportion of subjects with hypertension increased according to the severity of obesity.

**Table 1:** Baseline characteristics of patients with/without hemorrhagic transformation

	**Hemorrhagic transformation**		*P *value*
	**Absent (n = 306)**	**Present (n = 59)**	
Demographic			
Age, y	64.4 ± 12.4	66.3 ± 9.1	0.28
Gender, male	209 (68.3%)	37 (62.7%)	0.45
Body-mass index, kg/m^2^†	24.2 ± 3.2	23.1 ± 4.2	0.02
Clinical			
Hypertension	182 (59.3%)	37 (62.7%)	0.67
Diabetes	93 (30.4%)	17 (28.8%)	0.88
Hyperlipidemia	47 (15.4%)	5 (8.5%)	0.22
Smoking			0.95
Never	192 (62.7%)	37 (62.7%)	
Past	51 (16.7%)	9 (15.3%)	
Current	63 (20.6%)	13 (22%)	
Previous use of antiplatelet agents	9 (2.9%)	2 (3.4%)	0.69
Previous use of anticoagulation	9 (2.9%)	2 (3.4%)	0.69
Initial NIHSS score, median (IQR)†	8 (4–16)	3 (2–6)	< 0.01
Acute treatment			
Thrombolysis†	10 (3.3%)	14 (23.7%)	< 0.01
Acute heparin treatment	98 (32%)	21 (35.6%)	0.65
Stroke subtype†			< 0.01
Large artery atherosclerosis	135 (44.1%)	11 (18.6%)	
Cardioembolism	81 (26.5%)	30 (50.8%)	
Undetermined	90 (29.4%)	18 (30.5%)	
Laboratory			
Glucose, mmol/L	6.5 ± 2.1	6.9 ± 2.3	0.14
Total cholesterol, mmol/L	4.6 ± 1.0	4.6 ± 1.0	0.64
Systolic blood pressure, mmHg	152 ± 25	153 ± 27	0.87
Diastolic blood pressure, mmHg	88 ± 15	90 ± 17	0.39
Prolonged PT/aPTT (%)	36 (11.8%)	8 (13.6%)	0.66
Radiological			
Advanced WMLs	76 (25.2%)	16 (28.1%)	0.63
Presence of microbleeds	68 (22.7%)	15 (26.3%)	0.61

Values are mean ± SD or number of participants (percentage).

NIHSS indicates National Institute of Health Stroke Scale; WMLs, white matter lesion; and IQR, Interquartile range.

*P values were obtained using the χ^2^ test for categorical data, and the Student t test for continuous data.

†*P* < 0.05.

**Table 2:** The proportions of clinical radiological variables by the severity of obesity

	**Underweight**	**Normal weight**	**Overweight**	**Obesity I**	**Obesity II**	*P *for trend*
	**(< 18.5 kg/m**^**2**^**)**	**(18.5-22.9 kg/m**^**2**^**)**	**(23.0-24.9 kg/m**^**2**^**)**	**(25.0-29.9 kg/m**^**2**^**)**	**(≥ 30 kg/m**^**2**^**)**	
**Variables (n = 365)**	**n = 18 (4.9%)**	**n = 107 (29.3%)**	**n = 94 (25.8%)**	**n = 134 (36.7%)**	**n = 12 (3.3%)**	
Demographic						
Age, y	71.1 ± 11.2	65.3 ± 12.7	65.2 ± 11.8	63.4 ± 11.1	61.0 ± 12.8	0.08
Gender, male	15 (83.3%)	73 (68.2%)	62 (66.0%)	88 (65.7%)	8 (66.7%)	0.67
Clinical						
Hypertension†	7 (38.9%)	50 (46.7%)	58 (61.7%)	96 (71.6%)	8 (66.7%)	< 0.01
Diabetes	3 (16.7%)	29 (27.1%)	32 (34.0%)	44 (32.8%)	2 (16.7%)	0.38
Hyperlipidemia	1 (5.6%)	13 (12.1%)	17 (18.1%)	20 (14.9%)	1 (8.3%)	0.55
Current smoking	7 (38.9%)	21 (19.6%)	23 (24.5%)	23 (17.2%)	2 (16.7%)	0.23
Initial NIHSS score†	5 (3–12)	5 (2–11)	4 (2–9)	3 (2–5)	3 (1–6)	< 0.01
Thrombolysis	0 (0%)	9 (8.4%)	6 (6.4%)	9 (6.7%)	0 (0%)	0.61
Acute heparin treatment	9 (50%)	37 (34.6%)	31 (33%)	39 (29.1%)	3 (25%)	0.44
Antiplatelet Use	0	5 (4.7%)	4 (4.3%)	2 (1.5%)	0	0.48
Warfarin Use	0	2 (1.9%)	5 (5.3%)	4 (3.0%)	0	0.54
Radiological						
Hemorrhagic transformation†	4 (22.2%)	26 (24.3%)	14 (14.9%)	14 (10.4%)	1 (8.3%)	0.04
Advanced WMLs	6 (35.3%)	26 (25%)	24 (26.1%)	35 (26.3%)	1 (8.3%)	0.6
Presence of microbleeds	2 (11.8%)	20 (19.4%)	21 (22.8%)	36 (27.1%)	4 (33.3%)	0.42

Values are mean ± SD, median (interquartile range), or number of participants (percentage).

NIHSS indicates National Institute of Health Stroke Scale; WMLs, white matter lesion.

*Based on χ^2^ test of trend across the severity of obesity.

†*P* < 0.05.

In the obese group, 15 (10.3%) subjects had HTf, and compared with the normal weight group, the crude OR for HTf in the obese group was 0.36 (95% CI, 0.18 to 0.71). After adjusting age, gender, hypertension, diabetes, hyperlipidemia, atrial fibrillation (reflecting stroke subtype), smoking, initial NIHSS score (reflecting stroke severity), thrombolytic and acute heparin treatment, and the presence of advanced WMLs and microbleeds, a 61% risk reduction of HTf in the obese group existed when compared with the normal weight group (Table 3). However, ORs for HTf or cerebral microbleeds did not decrease in the overweight or the obese groups compared with the normal group (Table 4).

**Table 3:** The risk of hemorrhagic transformation according to the severity of obesity

	**OR for HTf**	
	**Unadjusted OR (95% CI)**	Adjusted OR (95% CI)*
Underweight (<18.5 kg/m^2^)	0.89 (0.27-2.94)	1.05 (0.28-3.96)
Normal (18.5-22.9 kg/m^2^)	1.00 (reference)	1.00 (reference)
Overweight (23.0-24.9 kg/m^2^)	0.55 (0.27-1.12)	0.48 (0.21-1.11)
Obesity (≥25 kg/m^2^)	0.36 (0.18-0.71)†	0.39 (0.17-0.87)†

HTf indicated hemorrhagic transformation; OR, odds ratio; and CI, confidence interval.

*ORs (95% CI) were adjusted by age, gender, hypertension, diabetes, hyperlipidemia, current smoking, initial NIHSS score, thrombolysis, acute heparin treatment, stroke subtype, previous aspirin use, previous warfarin use, and the presence of advanced WMLs and cerebral microbleeds.

†*P* < 0.05.

**Table 4:** The risk of hemorrhagic transformation or cerebral microbleeds according to the severity of obesity

	**OR for HTf or cerebral microbleeds**	
	**Unadjusted OR (95% CI)**	Adjusted OR (95% CI)*
Underweight (<18.5 kg/m^2^)	0.71 (0.23-2.18)	0.65 (0.19-2.25)
Normal (18.5-22.9 kg/m^2^)	1.00 (reference)	1.00 (reference)
Overweight (23.0-24.9 kg/m^2^)	0.87 (0.48-1.57)	0.80 (0.42-1.52)
Obesity (≥25 kg/m^2^)	0.93 (0.55-1.57)	0.92 (0.51-1.69)

HTf indicated hemorrhagic transformation; OR, odds ratio; and CI, confidence interval.

*ORs (95% CI) were adjusted by age, gender, hypertension, diabetes, hyperlipemia, current smoking, initial NIHSS score, thrombolysis, acute heparin treatment, stroke subtype, previous aspirin use, previous warfarin use, and the presence of advanced WMLs.

## Discussion and Conclusions

In the present study on patients with first-ever acute ischemic stroke, we found that the occurrence of HTf decreased according to the severity of obesity. In particular, obese subjects had a significantly decreased risk of HTf when compared with the normal weight group, after adjusting possible confounders including acute and previous treatment, and stroke severity and subtype.

Although our finding may seem controversial, the results are not surprising. Considering HTf as poor prognostic factor [3], the paradoxical effect of obesity on death after ischemic stroke, the most deleterious outcome, has been reported. In a Danish cohort of hospitalized acute stroke patients, post-stroke mortality was inversely related to obesity [18]. The phenomenon was also noted in an analysis of a health and nutrition status survey in US individuals, which showed that in aged population, obese stroke survivors tend to have a lower risk of mortality than patients with a normal weight [19]. However, the mechanism, which states that obesity increases the longevity in obese patient after ischemic stroke is not clear. Based on our findings and a review of the previous studies, HTf might be related to “obesity paradox” in acute ischemic stroke.

In the present study it was observed that as increasing of obesity severity, the proportion of cardioembolic stroke and severe stroke (reflected by initial NIHSS score) decreased, despite of the elevated prevalence of hypertension [20]. The incidence of HTf may be influenced by the differences in these baseline characteristics among BMI groups [10]. To reduce these confounding effects, we conducted a multivariable analysis after adjusting various possible confounders including the nature of stroke (severity and subtype), acute and previous treatment. After this adjustment, the presence of obesity independently predicted the occurrence of HTf in our study. However, the association between microbleeds and obesity was not elucidated in this study with first-ever acute ischemic stroke patients. Although microbleeds were suggested as a risk factor for cerebral hemorrhage [21], they are not a confirmed mediator of hemorrhagic transformation after ischemic stroke. Compared with HTf, the weak relationship between obesity and microbleeds may reflect the different pathophysiology of HTf and cerebral microbleeds. While the abrupt reperfusion after ischemic tissues elicits HTf, the spontaneous rupture of injured small arterioles in brain leads cerebral microbleeds.

For the cause of bleeding-obesity paradox, biological explanations exist. First, alterations in circulating coagulation factors have been suggested. Obesity has been related to higher levels of multiple coagulation factors such as factor VII, VIII, fibrinogen, and plasminogen activator inhibitor-1 [22,23]. Second, in studies investigating the association between BMI and platelet aggregation, patients with BMI ≥ 25 kg/m^2^ were found to have suboptimal response to antithrombotics than patients of normal weight [24,25]. However, this phenomenon was evaluated in normal or coronary artery disease subjects, and not in acute stroke patients. To clearly explain the “bleeding-obesity paradox,” the assessment of the coagulability and drug response in obese patient with acute ischemic stroke is required.

In the present investigation, a number of points require further clarification. First, due to the limitation of study population, we focused on the relatively mild obese patients and did not evaluate the phenomenon of “bleeding-obesity paradox” in severely obese patients. To assess the effect of BMI as a whole from underweight to severe obese, we suggest that future studies on ischemic stroke patients with widely-spread BMI should be undertaken. Second, the long-term outcome of HTf, which is a major poor prognostic factor in acute ischemic stroke, was not evaluated in this study. Thus, we could not assess “obesity paradox” *per se* in this study. Third, more severe stroke patients were excluded because they could not undergo brain MRI or did not have clinical information such as height, and the effects of obesity on HTf in severe stroke patients might not be evaluated completely.

The present study is unique because it documents the better results about HTf in obese patients with acute ischemic stroke, which persists after adjustment for acute treatment and stroke severity and subtype. Thus, we suggest the existence of a “bleeding-obesity paradox” in ischemic stroke, and propose that the inverse association between HTf and obesity in ischemic stroke may be considered in the management of acute stroke patients.

## Competing interests

The authors declare that they have no competing interests.

## Authors’ contributions

CKK and S-HL devised the original study concept and design. BJK and W-SR participated in the acquisition of data. CKK and S-HL performed statistical analyses, interpreted the results and wrote the manuscript. All authors read and approved the final manuscript.

## Pre-publication history

The pre-publication history for this paper can be accessed here:

http://www.biomedcentral.com/1471-2377/13/123/prepub

## Supplementary Material

Additional file 1: Table S1 Baseline characteristics of excluded patients. Click here for file
