# Supplementary material for: Paradoxical effect of obesity on hemorrhagic transformation after acute ischemic stroke
Source: BMC Neurol. 2013 Sep 23;13:123. doi: 10.1186/1471-2377-13-123 (PMC3848776; doi:10.1186/1471-2377-13-123)
Supplement: Additional file 1: Table S1 — Baseline characteristics of excluded patients. [file 1471-2377-13-123-S1.docx]

**Supplemental Table S1. Baseline characteristics of excluded patients**

|  | **Included patients  (n = 365)** | **Excluded patients (n=70)** | ***P* value*** |
| --- | --- | --- | --- |
| **Demographic** |  |  |  |
| **Age, y** | **64.7 ± 11.9** | **64.3 ± 11.8** | **0.76** |
| **Gender, male** | **246 (67.4%)** | **42 (60%)** | **0.27** |
| **Body-mass index, kg/m^2^** | **24.1 ± 3.4** | **23.3 ± 2.9** | **0.10** |
| **Obesity** |  |  | **0.13** |
| **Underweight (<18.5 kg/m^2^)** | **18 (4.9%)** | **2 (3.6%)** |  |
| **Normal (18.5-22.9 kg/m^2^)** | **107 (29.3%)** | **25 (44.6%)** |  |
| **Overweight (23.0-24.9kg/m^2^)** | **94 (25.8%)** | **13 (23.2%)** |  |
| **Obesity (≥25 kg/m2)** | **146 (40%)** | **16 (28.6%)** |  |
| **Missing value** | **0** | **14** |  |
| **Clinical** |  |  |  |
| **Hypertension** | **219 (60%)** | **46 (65.7%)** | **0.42** |
| **Diabetes** | **110 (30.1%)** | **20 (28.6%)** | **0.89** |
| **Hyperlipidemia** | **52 (14.2%)** | **11 (15.7%)** | **0.71** |
| **Smoking** |  |  | **0.46** |
| **Never** | **229 (62.7%)** | **49 (70%)** |  |
| **Past** | **60 (16.4%)** | **8 (11.4%)** |  |
| **Current** | **76 (20.8%)** | **13 (18.6%)** |  |
| **Previous antiplatelet use** | **11 (3.0%)** | **1 (2.3%) (missing number: 27)** |  |
| **Previous warfarin use** | **11 (3.0%)** | **1 (2.3%) (missing number: 27)** |  |
| **Stroke subtype** |  |  | **0.07** |
| **Large artery atherosclerosis** | **146 (40%)** | **24 (34.3%)** |  |
| **Cardioembolism** | **111 (30.4%)** | **31 (44.3%)** |  |
| **Undetermined** | **108 (29.6%)** | **15 (21.4%)** |  |
| **Initial NIHSS score†** | **4 (2-8)** | **6 (2-17)** | **<0.01** |
| **In-hospital death†** | **3 (0.8%)** | **4 (5.7%)** | **0.02** |
| **Values are mean±SD, median (interquartile range), or number of participants (percentage). NIHSS indicates National Institute of Health Stroke Scale; WMLs, white matter lesion. **P* values were obtained using the χ2 test for categorical data, and the Student t test for continuous data. †*P* < 0.05.** | | | |
